# Overt hypothyroidism complicated by complete heart block and severe hyperlipidemia. A case report

**DOI:** 10.1016/j.amsu.2022.104830

**Published:** 2022-11-06

**Authors:** Mohamud Mire Waberi, Said abdirahman, Mesut Karataş, Lütfi Öcal, Mohmed Omar Hassan, Ikram Awad, Mohamed Sheikh Hassan

**Affiliations:** a Department of Cardiology, Mogadishu Somali Turkish Training and Research Hospital, Mogadishu, Somalia; b Admas University, Borama, Somalia; c Department of Neurology, Mogadishu Somali Turkish Training and Research Hospital, Mogadishu, Somalia

**Keywords:** Hoypothyriodism, Complete atrioventriculer block, Dyslipidemia, Thyroxine

## Abstract

**Background:**

Hypothyroidism can cause a variety of manifestations, including cardiovascular disorders. The most frequent clinical signs are sinus bradycardia and pericardial effusion. The affected patient generally has significant symptoms. Hypothyroidism infrequently results in a complete atrioventricular block.

**Case presentation:**

A 19-year-old girl presented to our cardiology clinic with generalized tiredness, edema in her lower limbs and face, constipation, and a menstruation abnormality in the previous six months. With a normal ejection fraction on echocardiography, an electrocardiogram revealed complete atrioventricular block. When she was admitted, her laboratory testing showed that she had severely raised Thyroid Stimulating Hormone (TSH) levels, severe dyslipidemia with normal electrolytes, and normal liver and kidney function tests. The patient was treated with 50mg Thyroxine to her. She had significant improvement within two weeks of treatment. Up on the next follow-up (at one-month), her electrocardiogram returned to normal sinus rhythm without any evidence of atrioventricular block and that the lipid profile had returned to normal.

**Clinical discussion:**

In its first stages, hypothyroidism can not show any obvious symptoms. Untreated hypothyroidism over time can lead to a variety of health issues, including obesity, joint discomfort, infertility, and heart disease. This current case demonstrates how levothyroxine medication successfully managed a young female patient's severe hypothyroidism, difficult total heart block, severe hyperlipidemia, and long-standing menstrual irregularity.

**Conclusion:**

We found that overt hypothyroidism caused a complete atrioventricular block and severe dyslipidemia, and that thyroxin therapy completely corrected both conditions.

## Highlights

• We have seen a literature review the importance of finding out possible underlying cause of.complete heart block before intervention.
• Thyroid dysfunction can cause severe complication like complete heart block,dyslipidaemia and menstrual dysregulation.
• According to reports, levothyroxine alone can correct both av block and dyslipidaemia in the setting of hypothyroidism.
• Here we present a case of severe hypothyroidism complicating complete heart block and hyperlipidemia which was successfully treated with Levothyroxine.

## 1 Introduction

Several pathologic disorders that impair the structure or function of the conduction system might result in complete AV block. Complete AV block is usually brought on by cardiac conditions such as ischemic heart disease or cardiomyopathies, degenerative disorders characterized by escalating conduction system fibrosis, and medications that interfere with AV conduction. An AV block can also be caused by thyroid conditions or an electrolyte imbalance, particularly hyperkalemia^.^ [1]^.^

Hypothyroidism can cause a variety of manifestations, including cardiovascular disorders. The most frequent clinical signs are sinus bradycardia and pericardial effusion. The affected patient generally has significant symptoms. Hypothyroidism infrequently results in a complete atrioventricular block^.^ [2]^.^ Electrographic abnormalities linked to hypothyroidism include sinus bradycardia, flattened P waves, flat or inverted T waves, low voltage, and delayed intraventricular conduction [3]^.^

There are two different types of dyslipidemia: primary and secondary. Single or several gene mutations result in either an excess of triglycerides (TG) and low-density lipoprotein (LDL) production or a deficiency in their clearance, or an excess of high-density lipoprotein (HDL) production or clearance (HDL). Secondary dyslipidemia may result from medications, hypothyroidism, or other underlying disorders. In between 30 and 40% of all cases of dyslipidemia, are secondary dyslipidemia [4]^.^ We present a case of severe dyslipidemia and complete heart block brought on by severe hypothyroidism. Thyroid hormone replacement therapy was subsequently used to treat both conditions.

## 2 Case presentation

A 19-year-old female visited our cardiology clinic with a history of fatigue and facial swelling, she also complained of constipation and dizziness. as well as a long-standing history of menstrual irregularities. She had no known chronic disease as well as a family history of cardiac disease. The basic vital signs were unremarkable except for the heart rate of 45 beats per minute.

**Fig. 1:**
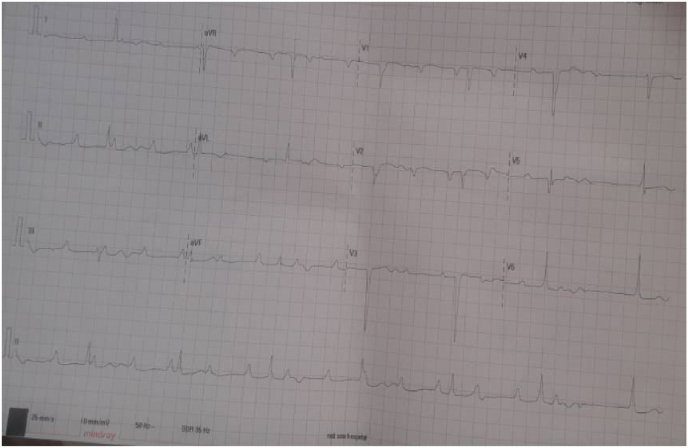
**1**^**st**^Electrocardiogram showing complete heart block with heart rate of 40 b.p.m.

On examination, she was alert with a swollen face, and swollen thyroid could not be found on the neck. The jugular venous pressure was normal. Cardiac auscultation indicated normal heart sounds with no added sounds. The lungs were clear on auscultation. Systemic reviews were unremarkable except for grade two bi-lateral non-pitting lower limb edema. An electrocardiogram showed complete atrio-ventricular block, atrioventricular dissociation, and a maximum heart rate of 42 beats/min (see Fig. 1). The echocardiography showed a normal ejection fraction.

**Fig. 2:**
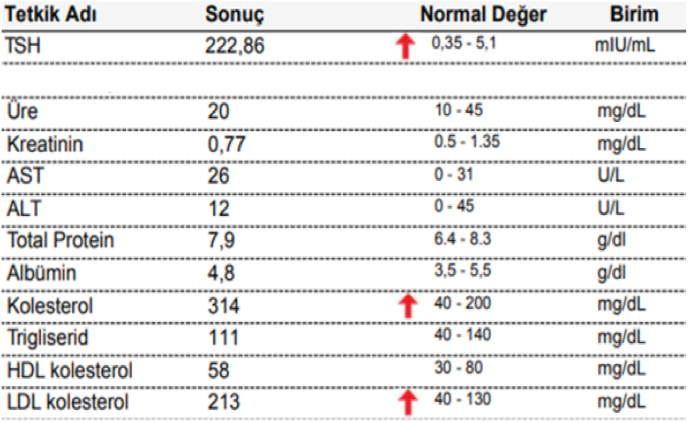
1st blood test demonstrating elevated lipid profile (cholesterol and LDL**)** and TSH (red arrow). (For interpretation of the references to colour in this figure legend, the reader is referred to the Web version of this article.)

Basic laboratory tests such as full blood count, thyroid, renal, and liver function tests were requested to rule out anemia, hypothyroidism, and renal and liver failure with normal results except for severe elevated Thyroid Stimulating Hormone levels (213 mIU/ml), and LDL levels (198 mg/dl) (see Fig. 2)**.** The patient was managed with 50mg of levothyroxine.

**Fig. 3:**
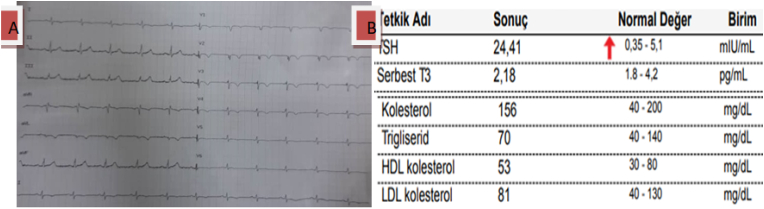
**A: 2**nd Electrocardiogram resolution showing complete heart block. **B: 2**^**nd**^lab tests showing improvement of Lipid profile with declining of TSH.

A follow-up of 1 month later, there was no further complaint with menstrual cycle normalization, while the electrocardiogram showed improved heart rate without any atrioventricular block and marked reduction of TSH and lipid profiles (see Fig. 3A&B). The dose of levothyroxine was reduced to 25mg with further follow-up. This case has been reported in line with the SCARE 2020 criteria [19].

## 3 Discussion

In a variety of clinical circumstances, conduction block from the atrium to the ventricle can develop for a variety of reasons. There are two types of etiologies: functional and structural. Functional diseases (metabolic illnesses, including hypothyroidism) are described as reversible in internal medical textbooks [5]. Hypothyroidism is marked by a reduction in oxygen and substrate use by the body's entire major organ systems. Hypothyroidism causes a decrease in cardiac output and contractility, as well as a drop in heart rate, accelerated atherosclerosis, and an increase in vascular resistance [6].

Thyroid hormones are also involved in the generation and conduction of electrical impulses in the heart. Changes in the cardiovascular system might cause the presenting symptom in individuals with hypothyroidism and hyperthyroidism. Cardiovascular dysfunction symptoms are infrequent in hypothyroid people. Exertional dyspnea, cold intolerance, and constipation and menstrual abnormalities are common symptoms. Some of the physical examination findings include bradycardia, hypertension (diastole), non-pitting edema, and pleural or pericardial effusion [7]^.^

The frequency of hypothyroidism in healthy elderly people ranges from 0.55 to 1.5%^.^ [8,9] There are a few case reports in the literature of improved AV block with the normalization of TSH levels following hypothyroidism therapy. Schoenmakers et al. documented an older woman who had total AV block as a result of severe hypothyroidism, which was resolved with levothyroxine treatment [10]^.^ A case report of reversible AV block in combination with myxedema was also published in the literature [11]^.^

Reversible AV block has been linked to not just overt hypothyroidism but also subclinical hypothyroidism. A middle-aged man with temporary 2/1 AV block and subclinical hypothyroidism was described by Nakayama et al. A conduction abnormality was fully resolved after 1 month of thyroxine administration [12]^.^

Complete AV block associated with thyrotoxicosis has been seen in patients with additional risk factors such as viral diseases, rheumatic fever, hypercalcemia, or digitalis medication. Although the primary injury generally causes a complete heart block, thyrotoxicosis can exacerbate the disease. Certain individuals with AV block, on the other hand, have been characterized in the literature as having thyrotoxicosis as their main underlying pathology, and thyroid disease therapy has restored normal AV conduction in these cases [13]^.^

Hyperlipidemia and hypothyroidism are associated with changes in lipid production, absorption, circulation, and metabolism. The liver's HMG-CoA reductase is expressed more often by thyroid hormones, which increases the production of cholesterol [14]^.^ Hypothyroidism reduces the production of hepatic cholesterol. However, this impact was overwhelmed by two other concurrent mechanisms. First, the Niemann-Pick C1-like 1 protein, a target of the lipid-lowering drug ezetimibe, causes an increase in gastrointestinal cholesterol absorption [15]^.^

Second, there is a reduction in the number of cell-surface LDL-cholesterol receptors, presumably as a result of T3-mediated effects on SREBP-2, which results in a decrease in plasma LDL-cholesterol clearance and an increase in apo-B lipoproteins [16]^.^ Observational studies confirm that among patients with overt hypothyroidism, 30% have increased total cholesterol and LDL levels, and 90% have dyslipidemia. Additionally, levothyroxine therapy restores lipid changes, with the exception of people who have underlying hyperlipidemia [17]^.^

Dyslipidemia is a recognized entity in hypothyroidism (both subclinical and overt hypothyroidism), and levothyroxine replacement therapy has a variety of positive effects on lipid markers in both subclinical and overt hypothyroidism patients. Levothyroxine treatment led to reversal to normal in more than 80% of subclinical hypothyroidism cases linked with hypercholesterolemia; comparable reversal is also reported in hypertriglyceridemia but in a lower number of cases^.^ [18]^).^ Levothyroxine therapy went well for our patient, as planned, and a permanent pacemaker and statin supplement were avoided.

## 4 Conclusion

We conclude by reporting a case of a patient who had severe dyslipidemia and a complete heart block in the setting of thyroid dysfunction. This case highlights the need to identify reversible causes of AV block prior to unnecessary pacemaker placement and statin medication.

## Ethical approval

According to our hospital rule, Ethical approval is only required in articles but not case reports.

## Sources of funding

There is no funding source for this study.

## Author contributions

All authors contributed toward writing, analysis, drafting, and revising the paper and they gave final approval of the version to be published, and agree to be accountable for all aspects of the work.

## Registration of research studies

Name of the registry: Not applicable
Unique Identifying number or registration ID: Not applicable
Hyperlink to your specific registration (must be publicly accessible and will be checked): Not applicable

## Consent

Written informed consent was obtained from the patient for publication of this case report and accompanying images.

## Guarantor

Mohamud Mire Waberi.

## Availability of data and materials

N/A.

## Provenance and peer review

Not commissioned, externally peer reviewed.

## Declaration of competing interest

I declare that there is no competing interest related to the study, authors, other individuals, or organizations.
